# Ocean Acidification Reduces Growth and Calcification in a Marine Dinoflagellate

**DOI:** 10.1371/journal.pone.0065987

**Published:** 2013-06-11

**Authors:** Dedmer B. Van de Waal, Uwe John, Patrizia Ziveri, Gert-Jan Reichart, Mirja Hoins, Appy Sluijs, Björn Rost

**Affiliations:** 1 Marine Biogeosciences, Alfred Wegener Institute for Polar and Marine Research, Bremerhaven, Germany; 2 Department of Aquatic Ecology, Netherlands Institute of Ecology (NIOO-KNAW), Wageningen, The Netherlands; 3 Ecological Chemistry, Alfred Wegener Institute for Polar and Marine Research, Bremerhaven, Germany; 4 Institute of Environmental Science and Technology (ICTA), Universitat Autònoma de Barcelona, Barcelona, Spain; 5 Department of Earth Sciences, Vrije Universiteit Amsterdam, Amsterdam, The Netherlands; 6 Department of Earth Sciences, Utrecht University, Utrecht, The Netherlands; 7 Geology Department, Royal Netherlands Institute for Sea Research (NIOZ), Den Hoorn (Texel), The Netherlands; Institute of Marine Research, Norway

## Abstract

Ocean acidification is considered a major threat to marine ecosystems and may particularly affect calcifying organisms such as corals, foraminifera and coccolithophores. Here we investigate the impact of elevated *p*CO_2_ and lowered pH on growth and calcification in the common calcareous dinoflagellate *Thoracosphaera heimii*. We observe a substantial reduction in growth rate, calcification and cyst stability of *T. heimii* under elevated *p*CO_2_. Furthermore, transcriptomic analyses reveal CO_2_ sensitive regulation of many genes, particularly those being associated to inorganic carbon acquisition and calcification. Stable carbon isotope fractionation for organic carbon production increased with increasing *p*CO_2_ whereas it decreased for calcification, which suggests interdependence between both processes. We also found a strong effect of *p*CO_2_ on the stable oxygen isotopic composition of calcite, in line with earlier observations concerning another *T. heimii* strain. The observed changes in stable oxygen and carbon isotope composition of *T. heimii* cysts may provide an ideal tool for reconstructing past seawater carbonate chemistry, and ultimately past *p*CO_2_. Although the function of calcification in *T. heimii* remains unresolved, this trait likely plays an important role in the ecological and evolutionary success of this species. Acting on calcification as well as growth, ocean acidification may therefore impose a great threat for *T. heimii*.

## Introduction

The oceans have taken up about one third of all CO_2_ emitted by anthropogenic activities since the onset of the industrial revolution [1]–[3]. This directly impacts seawater carbonate chemistry by increasing concentrations of CO_2_ and bicarbonate (HCO_3_^−^), decreasing concentrations of carbonate (CO_3_^2−^) and a lowering of pH [4]. The acidification of ocean waters might impact marine life, notably calcifying organisms that use inorganic carbon to produce a calcium carbonate (CaCO_3_) shell. Calcifying organisms play an important ecological and biogeochemical role in marine ecosystems, evident from extensive coral reefs and vast calcite deposits found in geological records. Ocean acidification has been shown to reduce calcification of various key calcifying organisms such as corals [5], foraminifera [6], and coccolithophores [7], [8]. Little is yet known about the general responses of calcareous dinoflagellates [9], and no study so far investigated the impact of ocean acidification on their calcification.

Dinoflagellates feature a complex life-cycle that often includes formation of cysts. In some species, these cysts are made of calcite and can contribute substantially to the ocean carbonate flux in certain regions [10]–[12]. *Thoracosphaera heimii*, the most common calcareous dinoflagellate species in present-day ocean, is autotrophic and occurs typically in subtropical and tropical waters [13]–[15]. The main life-cycle stage of *T. heimii* comprises coccoid vegetative cells with a calcium carbonate shell, so-called vegetative cysts [16], [17]. Although the term cyst is most often used for long-term resting stages that are typically produced after sexual reproduction, in *T. heimii* this term is used for its coccoid vegetative stage. Cysts of *T. heimii* can be commonly found in the fossil record in sediments dating back to the Cretaceous [18]. Therefore, *T. heimii* cysts may serve as potential proxy for reconstructing the past climate. For instance, Sr/Ca ratios have been shown to correlate well with sea surface temperatures [19], but also the oxygen and carbon isotopes trapped in the cysts could provide useful proxies.

The oxygen isotopic composition (δ^18^O) of calcite was found to be strongly controlled by the temperature and the δ^18^O of the seawater in which the organism calcifies [20]–[22]. In abiotic precipitation experiments, the δ^18^O of calcite is mainly a function of the δ^18^O and speciation of dissolved inorganic carbon (DIC), where dissolved CO_2_ is heavier with respect to ^18^O than HCO_3_^−^ and CO_3_^2−^ [23], [24]. Similarly, the carbon isotopic composition (δ^13^C) of calcite is predominantly controlled by the δ^13^C and speciation of DIC, yet dissolved CO_2_ is depleted with respect to ^13^C relative to HCO_3_^−^ and CO_3_^2−^ [21], [25]. In unicellular calcifiers like coccolithophores and *T. heimii*, calcification occurs intracellularly in specialized vesicles [16], [26], [27]. Therefore, the inorganic carbon used for calcification by these organisms must be derived from the intracellular inorganic carbon (C_i_) pool. Consequently, changes in δ^18^O and δ^13^C of calcite should resemble changes in the intracellular C_i_ pool and may provide insights in the physiological processes underlying calcification and organic carbon production.

Comparable to coccolithophores, ocean acidification likely reduces calcification in *T. heimii* as well. Furthermore, increasing concentrations of CO_2_ are expected to alter the stable carbon and oxygen isotopic composition of *T. heimii* cysts. To test these hypotheses, we grew *T. heimii* at a range of CO_2_ levels and followed its responses in growth and calcification. Besides the assessment of δ^18^O and δ^13^C in *T. heimii* as a proxy, we use its isotopic composition as a tool to understand processes involved in organic carbon production and calcification. Transcriptomic analyses were applied to reveal mechanisms underlying the observed responses.

## Materials and Methods

### Experimental Set-up

Cells of *Thoracosphaera heimii* RCC1512 (formerly AC214; Roscoff Culture Collection) were grown as dilute batch cultures in 2.4 L air-tight borosilicate bottles. Population densities were kept low at all times (<1,300 cells mL^−1^) in order to keep changes in carbonate chemistry minimal (i.e. <3.5% with respect to DIC; Table S1). Filtered natural seawater (0.2 µm) was enriched with metals and vitamins according to the recipe for f/2-medium, except for FeCl_3_ (1.9 µmol L^−1^), H_2_SeO_3_ (10 nmol L^−1^), and NiCl_2_ (6.3 nmol L^−1^). The added concentrations of NO_3_^−^ and PO_4_^3−^ were 100 µmol L^−1^ and 6.25 µmol L^−1^, respectively. Cultures were grown at a light:dark cycle of 16∶8 h and an incident light intensity of 250±25 µmol photons m^−2^ s^−1^ provided by daylight lamps (Lumilux HO 54W/965, Osram, München, Germany). Bottles were kept at 15°C and placed on a roller table to avoid sedimentation. Prior to inoculation, the culture medium was equilibrated with air containing 150 µatm CO_2_ (∼Last Glacial Maximum), 380 µatm CO_2_ (∼present-day), 750 and 1400 µatm CO_2_ (future scenarios assuming unabated emissions). Each treatment was performed in triplicate.

### Sampling and Analyses

Prior to the experiments, cells were acclimated to the respective CO_2_ concentrations for at least 21 days, which corresponds to >7 cell divisions. Experiments were run for 8 days and included >3 cell divisions. Cell growth was monitored by means of triplicate cell counts daily or every other day with an inverted light microscope (Axiovert 40C, Zeiss, Germany), using 0.5–2 ml culture suspension fixed with Lugol’s solution (2% final concentration in mQ). Cell counts included determination of vegetative cysts, i.e. shells containing cell material, and empty shells. Because empty shells also contain inorganic carbon, the total number of cysts was used for estimating inorganic carbon quota, while only vegetative cysts were included in the growth rate estimations. From each biological replicate, growth rates were estimated by means of an exponential function fitted through the number of vegetative cysts over time, according to:
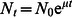
(1)where *N_t_* refers to the population density at time *t* (in days), *N_0_* to the population density at the start of the experiment, and *µ* to the growth rate (Fig. S1).

For total alkalinity (TA) analyses, 25 mL of culture suspension was filtered over glass-fibre filters (GF/F, ∼0.6 µm pore size, Whatman, Maidstone, UK) and stored in gas-tight borosilicate bottles at 3°C. Duplicate samples were analysed by means of potentiometric titrations using an automated TitroLine burette system (SI Analytics, Mainz, Germany). pH was measured immediately after sampling with a pH electrode (Schott Instruments, Mainz, Germany), applying a two-point calibration on the NBS scale prior to each measurement. For DIC analyses, 4 mL culture suspension was filtered over 0.2 µm cellulose-acetate filters, and stored in headspace free gas-tight borosilicate bottles at 3°C. Duplicate samples of DIC were analysed colorimetrically with a QuAAtro autoanalyser (Seal Analytical, Mequon, USA). Carbonate chemistry (Table S1) was assessed by total alkalinity (TA) in combination with pH_NBS_, temperature and salinity, using the program CO_2_sys [28]. For the calculations, an average phosphate concentration of 6.4 µmol L^−1^ was assumed, the dissociation constant of carbonic acid was based on Mehrbach et al. [29], refit by Dickson and Millero [30]. The dissociation constant of sulfuric acid was based on Dickson [31].

To determine the isotopic composition of DIC (δ^13^C_DIC_) and the water (δ^18^O_water_), 4 mL of culture suspension was sterile-filtered over 0.2 µm cellulose-acetate filters and stored at 3°C. Prior to analyses, 0.7 mL of sample was transferred to 8 mL vials. For determination of δ^13^C_DIC_, the headspace was filled with helium and the sample was acidified with three drops of a 102% H_3_PO_4_ solution. For determination of δ^18^O_water_, the headspace was flushed with helium containing 2% CO_2_. CO_2_ and O_2_ isotopic composition in the headspace were measured after equilibration using a GasBench-II coupled to a Thermo Delta-V advantage isotope ratio mass spectrometer with a precision of <0.1‰ [32].

At the end of each experiment, cultures were harvested for analyses of particulate organic carbon (POC) and related isotopic composition (δ^13^C_POC_), total particulate carbon (TPC), isotopic composition of the calcite (δ^13^C_calcite_ and δ^18^O_calcite_), and for the Scanning Electron Microscope (SEM). For POC and TPC analyses, 250–500 mL cell suspension was filtered over precombusted GF/F filters (12 h, 500°C) and stored at −25°C in precombusted Petri dishes. Prior to POC measurements, 200 µL of 0.2 N analytical grade HCl was added to the filters to remove all particulate inorganic carbon (PIC), and filters were dried overnight. POC, δ^13^C_POC_, and TPC were analysed in duplicate on an Automated Nitrogen Carbon Analyser mass spectrometer (ANCA-SL 20–20, SerCon Ltd., Crewe, UK). PIC was calculated as the difference in carbon content between TPC and POC. δ^13^C_calcite_ and δ^18^O_calcite_ were measured with a Thermo Scientific MAT253 coupled to a Kiel IV carbonate preparation device. Analytical stability and calibration was checked routinely by analyzing NBS19 and IAEA-CO1 carbonate standards. Reproducibility (Kiel IV and MAT253) was <0.05‰ and <0.03‰ for δ^18^O and δ^13^C, respectively.

For SEM analyses, 50 mL culture suspension was filtered over a 0.8 µm polycarbonate filter and dried overnight at 60°C. Filters were fixed on aluminium stubs, sputter-coated with gold-palladium using an Emscope SC500 Sputter Coater (Quorum Technologies, Ashford, UK), and viewed under a FEI Quanta FEG 200 scanning electron microscope (FEI, Eindhoven, the Netherlands). From each replicate, a total of >200 cysts were counted and assessed as complete or incomplete.

### Isotopic Fractionation

Isotopic fractionation during organic carbon production and calcification was calculated based on the carbon isotopic composition of the cellular organic carbon, cellular inorganic carbon and DIC, and the oxygen isotopic composition of the calcite and seawater, respectively. The carbon isotopic composition is reported relative to the PeeDee belemnite standard (PDB):
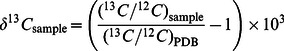
(2)


The isotopic composition of CO_2_ (δ^13^C_CO2_) was calculated from δ^13^C_DIC_ using a mass balance relation according to Zeebe and Wolf-Gladrow [24], applying fractionation factors between CO_2_ and HCO_3_^−^ from Mook et al. [33] and between HCO_3_^−^ and CO_3_^2−^ from Zhang et al. [34]. The isotopic fractionation during POC formation (ε_p_) was calculated relative to δ^13^C_CO2_ according to Freeman and Hayes [35]:
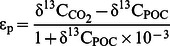
(3)


The carbon isotopic fractionation during calcite formation (ε_k_) was calculated relative to δ^13^C_DIC_:
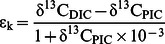
(4)


The oxygen isotopic composition in the calcite is also reported relative to the PDB standard:
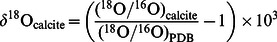
(5)


The oxygen isotopic composition in DIC (δ^18^O_DIC_) was determined using the oxygen fractionation factor between DIC, calculated after Zeebe and Wolf-Gladrow [24], and water (α_(DIC-H2O)_), calculated after Zeebe [36], with temperature corrected fractionation factors from Beck et al. [37]. The isotopic composition of DIC (δ^18^O_DIC_) was calculated according to:
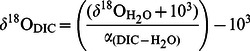
(6)


### Transcriptomic Analyses

For RNA extraction, 500 mL of culture suspension was concentrated to 50 mL with a 10 µm mesh-sized sieve, and subsequently centrifuged at 15°C for 15 min at 4000 g. Cell pellets were immediately mixed with 1 mL 60°C TriReagent (Sigma-Aldrich, Steinheim, Germany), frozen with liquid nitrogen and stored at −80°C. Subsequently, cell suspensions were transferred to a 2 mL cryovial containing acid washed glass beads. Cells were lysed using a BIO101 FastPrep instrument (Thermo Savant, Illkirch, France) at maximum speed (6.5 m s^−1^) for 2×30 s, with an additional incubation of 5 min at 60°C in between. For RNA isolation, 200 µL chloroform was added to each vial, vortexed for 20 s and incubated for 10 min at room temperature. The samples were subsequently centrifuged for 15 min at 4°C with 12,000 g. The upper aqueous phase was transferred to a new vial and 2 µL 5 M linear acrylamide, 10% volume fraction of 3 M sodium acetate, and an equal volume of 100% isopropanol were added. Mixtures were vortexed and subsequently incubated overnight at −20°C in order to precipitate the RNA. The RNA pellet was collected by 20 min centrifugation at 4°C and 12,000 g. The pellet was washed twice, first with 70% ethanol and afterwards with 96% ethanol, air-dried and dissolved with 100 µl RNase free water (Qiagen, Hilden, Germany). The RNA sample was further cleaned with the RNeasy Kit (Qiagen) according to manufacturer’s protocol for RNA clean-up including on-column DNA digestion. RNA quality check was performed using a NanoDrop ND-100 spectrometer (PeqLab, Erlangen, Germany) for purity, and the RNA Nano Chip Assay with a 2100 Bioanalyzer (Agilent Technologies, Böblingen, Germany) was performed in order to examine the integrity of the extracted RNA. Only high quality RNAs (OD_260_/OD_280_>2 and OD_260_/OD_230_>1.8) as well as RNA with intact ribosomal peaks (obtained from the Bioanalyzer readings) were used for microarrays.

454-libraries were constructed by Vertis Biotechnologie AG (http://www.vertis-biotech.com/). From the total RNA samples poly(A)+ RNA was isolated, which was used for cDNA synthesis. First strand cDNA synthesis was primed with an N6 randomized primer. Then 454 adapters were ligated to the 5′ and 3′ ends of the cDNA, and the cDNA was amplified with 19 PCR cycles using a proof reading polymerase. cDNA with a size range of 500–800 bp was cut out and eluted from an agarose gel. The generated libraries were quantified with an RL-Standard using the QuantiFluor (Promega, Mannheim, Germany). The library qualities were assessed using the High Sensitivity DNA chip on the Agilent 2100 Bioanalyzer (Agilent, Waldbronn, Germany). For all sequencing runs 20×10^7^ molecules were used for the emulsion PCR that were carried out on a MasterCycler PCR cycler (Eppendorf, Hamburg, Germany). The following enrichment was performed according to the manufacturer’s instructions. Sequencing was performed with the GS Junior Titanium Sequencing Kit under standard conditions. The 454 Sequencing System Software version 2.7 was used with default parameters, i.e., Signal Intensity filter calculation, Primer filter, Valley filter, and Base-call Quality Score filter were all enabled.

### Statistical Analysis

Normality was confirmed using the Shapiro-Wilk. Variables were log-transformed if this improved the homogeneity of variances, as tested by Levene’s test. Significance of relationships between variables and concentration of CO_2_ and CO_3_^2−^ were tested by means of linear regression. Significance treatments was tested using one-way ANOVA, followed by post hoc comparison of the means using Tukey’s HSD (α = 0.05) [38].

## Results

**Figure 1:**
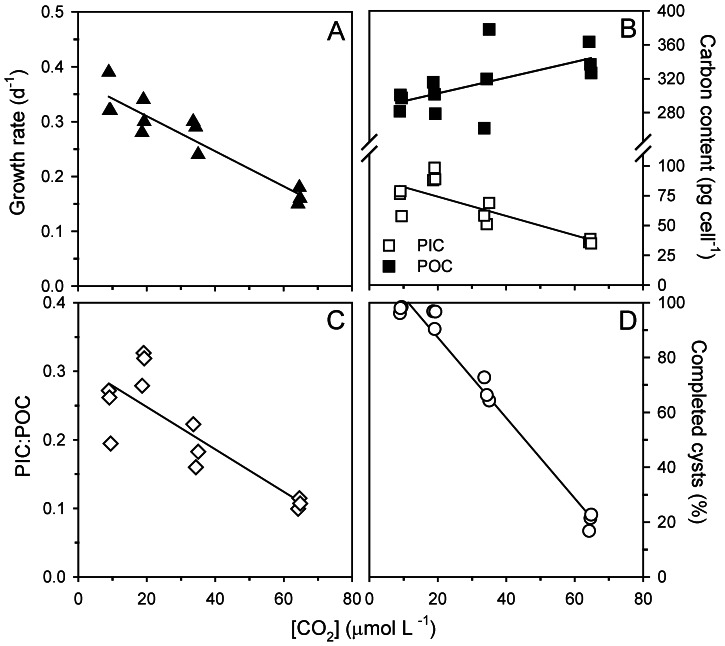
Effect of increasing CO_2_ concentrations on growth and calcification. (A) Specific growth rate, (B) PIC and POC, (C) PIC:POC ratio, and (D) fraction of completed cysts. Solid lines indicate linear regressions (*n* = 12) with (A) R^2^ = 0.94, P<0.001, (B) POC: R^2^ = 0.35, P = 0.042, and PIC: R^2^ = 0.66, P = 0.001, (C) R^2^ = 0.70, P<0.001, and (D) R^2^ = 0.98, P<0.001.

Increasing concentrations of CO_2_ cause a strong decline in growth (Fig. 1A), which decreases by up to 53% over the investigated CO_2_ range (Table S2). Although the total carbon quota (TPC) is not affected by CO_2_ (Table S2), the organic carbon quota (POC) gradually increases while the inorganic carbon quota (PIC) shows a substantial decrease (Fig. 1B). Consequently, the PIC:POC ratio strongly decreases with increasing concentrations of CO_2_ (Fig. 1C), showing a decrease of ∼54% from the lowest to the highest CO_2_ treatment (Table S2).

**Figure 2:**
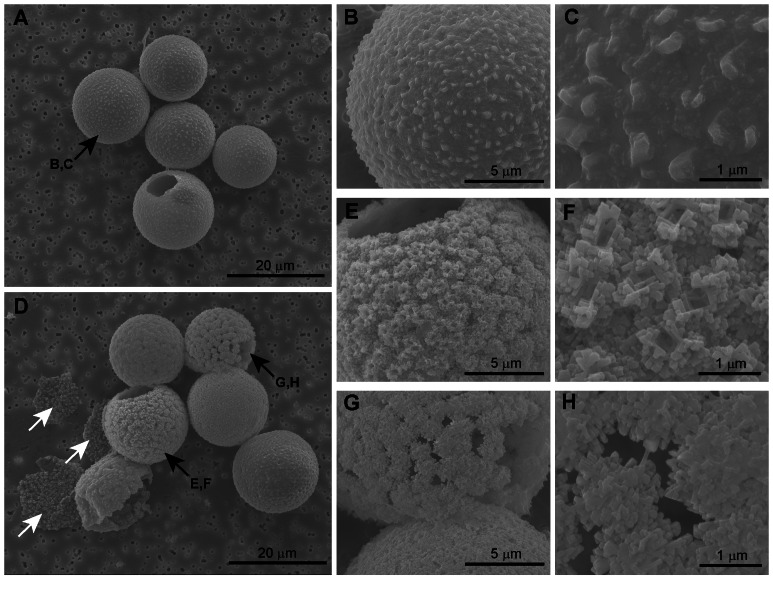
Effect of elevated *p*CO_2_ on cyst morphology. Cells grown under (A–C) 150 µatm CO_2_ and (D–H) 1400 µatm CO_2_. Black arrows indicate cysts that are shown in detailed images, white arrows show collapsed cysts.

The reduced degree of calcification is also evident from the cyst morphology. In the lowest CO_2_ treatment, the majority of cysts shows a fully closed and completed calcite structure (Fig. 2A–C). At the highest CO_2_ concentration, however, calcification of most cysts is incomplete (Fig. 2D–H). Some cysts show initial stages of calcification, indicated by typical square pores (Fig. 2E,F) [16]. In other cysts, the numerous crystallization sites remain unconnected showing clear cavities in the calcite structure (Fig. 2G,H). These cavities likely cause the collapse of many cysts upon filtration (Fig. 2D, white arrows). With increasing concentrations of CO_2_, the number of completed cysts dramatically decreases from ∼98% at the lowest CO_2_ treatment towards ∼18% at the highest CO_2_ treatment (Fig. 1D).

**Figure 3:**
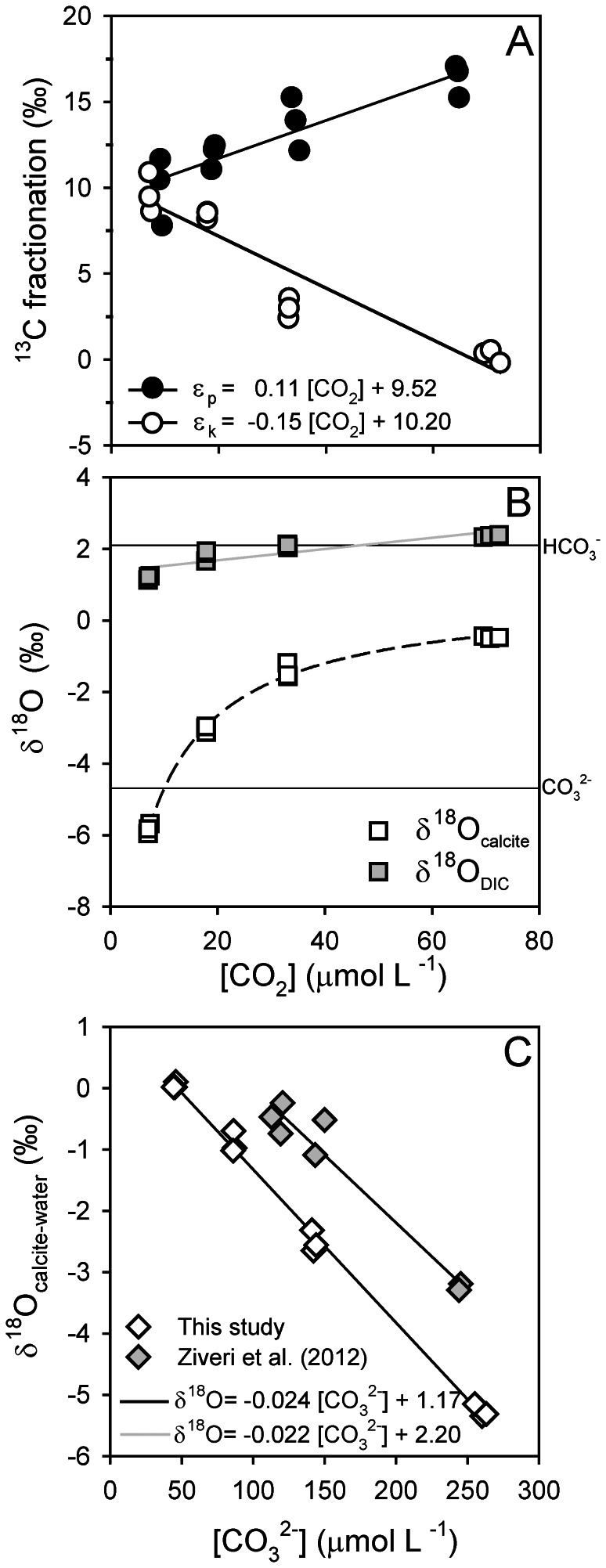
Effect of increasing CO_2_ concentrations on the stable isotope composition. (A) ^13^C fractionation of organic carbon (ε_p_) and calcite (ε_k_), (B) ^18^O composition of calcite (δ^18^O_calcite_) and DIC (δ^18^O_DIC_), and (C) relationship between the oxygen isotopic composition of calcite (δ^18^O_calcite-water_) in *Thoracosphaera* from this study (open diamonds) and from Ziveri et al. [9] (grey diamonds). Horizontal lines in (B) indicate δ^18^O values for HCO_3_^−^ and CO_3_^2−^, and dashed line indicates trend of curve. Solid lines indicate linear regressions (*n* = 12) with (A) ε_p_: R^2^ = 0.75, P<0.001, and ε_k_: R^2^ = 0.90, P<0.001, (B) δ^18^O_DIC_: R^2^ = 0.76, P<0.001, and (C) This study: R^2^ = 0.99, P<0.001, and Ziveri et al. (2012), (*n* = 7): R^2^ = 0.95, P<0.001.

Carbon isotope fractionation responds strongly to the applied CO_2_ treatments, showing an increase in ε_p_ and a decrease in ε_k_ with increasing *p*CO_2_ (Fig. 3A). In other words, the organic carbon fraction of the cells becomes depleted in ^13^C while the inorganic carbon fraction (i.e. the calcite) increases its ^13^C content. Furthermore, the calcite also becomes ^18^O-enriched, indicated by the increase in δ^18^O_calcite_ with increasing *p*CO_2_ (Fig. 3B). As dissolved CO_2_ is heavier than HCO_3_^−^ and CO_3_^2−^ [24], increasing CO_2_ levels cause δ^18^O_DIC_ to increase (Fig. 3B). Yet, changes are relatively small and the δ^18^O_DIC_ remains close to that of HCO_3_^−^, which is the dominant inorganic carbon species. To permit comparison with previous findings, δ^18^O_calcite_ values were corrected for the δ^18^O of water (−0.52±0.07 ‰) and plotted as a function of CO_3_^2−^ concentration (Fig. 3C). Calcite δ^18^O decreases strongly with increasing concentrations of CO_3_^2−^, and the slope is similar to the one reported for another *T. heimii* strain (RCC1511) [9].

The transcriptome indicates substantial gene regulation in response to changes in carbonate chemistry, with a total of 9701 genes being expressed (Fig. S2). The expression of the majority of genes was treatment specific, amounting to 3183, 2704, and 2176 genes in the low, present-day and high CO_2_ treatments, respectively (Fig. S2). Interestingly, the number of expressed genes to which a function could be assigned by comparison with public databases was highest in the low and present-day CO_2_ treatment (∼22%), and lowest in the high CO_2_ treatment (∼13%). The expressed genes from each treatment are differentially distributed over different ‘eukaryotic orthologous groups’ (KOGs; Fig. S3 and Table S3). Although the total number of expressed genes is largely comparable between treatments, different sets of genes within the KOGs are expressed. About 55% of the number of expressed and annotated genes in each treatment are associated to the KOGs ‘Translation, ribosomal structure and biogenesis’, ‘Signal transduction mechanisms’, ‘Posttranslational modification, protein turnover and chaperons’, and ‘Energy production and conversion’ (Fig. S3). Expression of genes associated to the latter two categories increased in response to increasing *p*CO_2_. In contrast, expression of genes involved in ‘Inorganic ion transport and metabolism’ decreased in the high CO_2_ treatment (Fig. S3).

**Figure 4:**
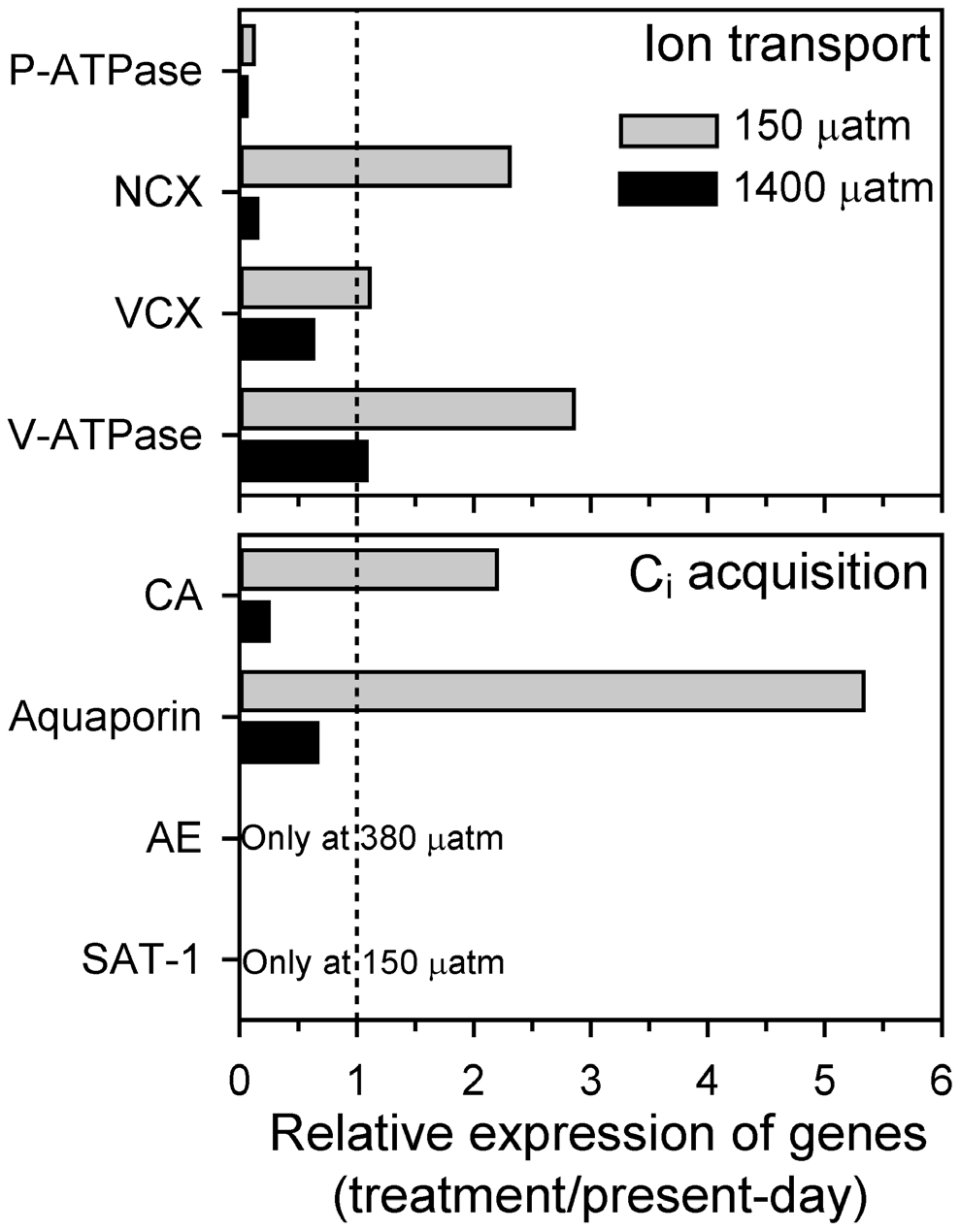
Effect of elevated *p*CO_2_ on gene regulation. Number of readings found for genes associated to ion transport and C_i_ acquisition in the 150 µatm and 1400 µatm CO_2_ treatments relative to the present-day (380 µatm) CO_2_ treatment.

We therefore investigated the genes involved in ion transport and inorganic carbon acquisition in more detail (Fig. 4; Table S4). We observed a substantial regulation of genes associated to vacuolar Ca^2+^ and H^+^ transport, including P-type Ca^2+^ ATPases, Ca^2+^/Na^+^ exchangers (NCX1), Ca^2+^/H^+^ antiporters (VCX), and vacuolar H^+^ ATPases (V-ATPase). In particular, the relative expression of genes associated to NCX and V-ATPase decreases from the low to the high CO_2_ treatment (Fig. 4). Similarly, the relative expression of genes associated to carbonic anhydrases (CA) and aquaporins decreases with increasing *p*CO_2_. In the present-day CO_2_ treatment, we observed expression of a gene associated to an SLC4 family anion exchanger (AE), most likely responsible for the transport of HCO_3_^−^ into the cell (Fig. 4) [39]. This gene was expressed in neither the low nor the high CO_2_ treatment. An SLC26 family SO_4_^3−/^HCO_3_^−/^C_2_O_4_^2−^ anion exchanger (SAT-1) was yet another exclusive expression of a gene only found in the low CO_2_ treatment. The potential role of this anion exchanger in C_i_ acquisition by phytoplankton remains to be elucidated.

## Discussion

### Growth and Carbon Production

Our results show considerable impacts of elevated *p*CO_2_ on *T. heimii*, with strong decreases in its growth rate and degree of calcification (Fig. 1, 2). Despite the increase in organic carbon quota (POC), the overall biomass production decreases substantially with increasing *p*CO_2_ (Table S2). Higher availability of CO_2_ has been shown to promote phytoplankton growth and carbon production [40], [41]. Such CO_2_ responses are typically associated to the poor catalytic properties of RubisCO, which is characterized by low affinities for its substrate CO_2_. Increasing concentrations of CO_2_ are however accompanied by a reduction in pH, which may have consequences for calcification. For the most common coccolithophore *Emiliania huxleyi*, lowered pH in fact hampers calcification while elevated *p*CO_2_ stimulates biomass production, causing a reallocation of carbon and energy between these key processes [42], [43]. This flexibility may explain why growth in *E. huxleyi* is typically not affected by ocean acidification [44]. In *T. heimii*, however, we observed a strong decrease in calcification, in biomass production as well as in growth. Apparently, *T. heimii* lacks the ability to efficiently reallocate cellular carbon between pathways and maintain growth relatively unaffected. Our data furthermore suggests that calcification plays a fundamental role in its growth, life cycle and hence survival. Recent findings have shown that growth and calcification by *E. huxleyi* may, at least partly, recover from ocean acidification as result of evolutionary adaptation [45]. Whether or not *T. heimii* exhibits such capabilities of adaptive evolution can only be answered from long-term incubations over hundreds of generations [46].

Transcriptomic analyses reveal a substantial regulation of genes in response to elevated *p*CO_2_. Even though no major shift in the relative distribution of expressed genes to the functional categories (KOGs) is induced by the treatments, *T. heimii* uses different sets of genes within these categories. There is a slight increase in the expression of genes associated to signal transduction and posttranslational modifications upon elevated *p*CO_2_, and a decrease in the expression of genes involved in inorganic ion transport (Fig. S3), suggesting that *T. heimii* readjusts its transcriptome on several levels when grown under different *p*CO_2_. Many phytoplankton species have the ability to deal with changes in CO_2_ availability by regulating their so-called carbon concentrating mechanism (CCMs) [47]–[49]. *T. heimii* also appears to regulate its proteome towards changes by down-regulating genes involved in CA and aquaporins under elevated *p*CO_2_, and by up-regulating these genes under lowered *p*CO_2_ (Fig. 4). CA accelerates the equilibrium between CO_2_ and HCO_3_^−^, and can be located both intra- and extracellularly. From our results it remains unclear whether *T. heimii* expresses intra- or extracellular CA. Yet, in both cases CA plays a key role in the CCM, as it replenishes the CO_2_ around RubisCO (intracellular) or the carbon source being depleted in the boundary layer due to active uptake (extracellular) [49], [50]. Aquaporins have been suggested to play a role in CO_2_ transport [47], [51], which is supported by the observed CO_2_-dependency in our expression patterns. Besides CO_2_ also HCO_3_^−^ is often transported into the cell, which will facilitate the high intra-cellular CO_2_ requirements imposed by RubisCO. Indeed, *T. heimii* expresses genes associated to putative HCO_3_^−^ transporters at both low and present-day *p*CO_2_, but not at high *p*CO_2_ (Fig. 4). Our results thus suggest a down-scaling of the CCM in *T. heimii* under elevated *p*CO_2_, which possibly makes energy available for other processes as it has been observed in other species [43], [52]. Yet it seems that neither the down-scaling of the CCM nor an extensive regulation of the transcriptome can compensate for the adverse effects of elevated *p*CO_2_ on growth and calcification in *T. heimii*.

### Calcification and Isotope Fractionation

Calcification in *T. heimii* was strongly affected by elevated *p*CO_2_. Along with a reduction in the degree of calcification (Fig. 1B,C), also the morphology of *T. heimii* cysts was influenced (Fig. 2). With elevated *p*CO_2_ the number of completed cysts dramatically decreased and the number of collapsed cysts increased. The completed calcite structures predominant at low and present-day *p*CO_2_ resemble those of mature *T. heimii* cells, whereas the incomplete calcite structures, prevailing under high *p*CO_2_, resemble those of young cells [16], [26]. The incomplete cysts in our experiments, however, often contain an opening through which the cell has left for division, being indicative for mature cells. Thus, cells remained either in the cyst too short for completing the calcite structure, the calcite cyst was directly affected by the low pH of the water, and/or cells reduced their calcification rates. Since growth rates were strongly reduced upon elevated *p*CO_2_, it seems unlikely that cells remained in the cyst stage too short for completion of the cyst, as could be expected under enhanced growth rates. Although pH in our highest CO_2_ treatment was close to 7.6, the water still remained supersaturated with respect to calcite (i.e. an Ω_calcite_ >1.2; Table S2), and calcite dissolution seem unlikely to have caused the incompletion and cavities in the calcite structure (Fig. 2). Thus, the large number of affected *T. heimii* cysts at elevated *p*CO_2_ seems mainly to be a result of reduced calcification rates by the cells.

Calcification in *T. heimii* likely takes place intracellularly in vesicles [16], [26], comparable to coccolithophores [9], [27]. Hence, the inorganic carbon needed for calcification is obtained from the intracellular inorganic carbon pool (C_i_), which may deviate strongly from external conditions in terms of speciation as well as isotopic composition. We observed an increase of carbon isotope fractionation for organic carbon production (ε_p_), whereas it decreased for calcite formation (ε_k_) in response to elevated *p*CO_2_ (Fig. 3A). With a higher availability of CO_2_, more of the intracellular C_i_ pool may be replenished by CO_2_, which is depleted in ^13^C compared to HCO_3_^−^. Consequently, RubisCO can fractionate against an isotopically lighter C_i_ pool and thus better express its preference for lighter ^12^C, which could explain the increasing ε_p_. As a consequence, the intracellular C_i_ pool becomes enriched with ^13^C by so-called Rayleigh distillation, which *a priori* could explain the decrease in ε_k_. However, increased CO_2_ availability in combination with a reduced organic carbon production should lead to a lowered Rayleigh distillation, and in fact decrease the enrichment of ^13^C within the cell. Also, Rayleigh distillation should always feedback on CO_2_ fixation as well as CaCO_3_ precipitation, and thus cannot explain the opposing trends of fractionation in those processes.

**Figure 5:**
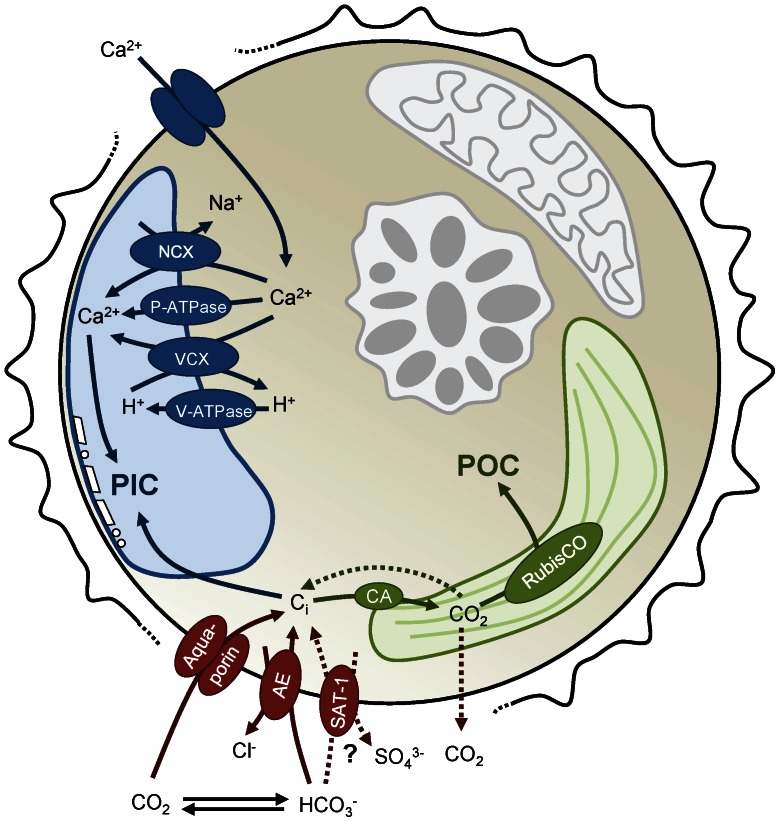
Conceptual model of regulated proteins in a *T. heimii* cell. The regulated proteins involved in ion transport and C_i_ acquisition are shown on their putative locations [39], [49], [57] **.** Proteins involved in vacuolar Ca^2+^ and H^+^ transport include P-type Ca^2+^ ATPases (P-ATPase), Ca^2+^/Na^+^ exchangers (NCX), Ca^2+^/H^+^ antiporters (VCX), and vacuolar H^+^ ATPases (V-ATPase). Active uptake of HCO_3_^−^ may occur via a SLC4 family anion exchanger (AE) or an SLC26 family SO_4_^3−/^HCO_3_^−/^C_2_O_4_^2−^ anion exchanger (SAT-1). Carbonic anhydrases (CA) are located intracellularly or extracellularly and enhance the interconversion between CO_2_ and HCO_3_^−^.

The opposing CO_2_ effects on ε_p_ and ε_k_ can thus only be explained if both processes use C_i_ pools that are isotopically different. CO_2_ fixation uses the C_i_ pool within the chloroplast, which is affected by the relative CO_2_ and HCO_3_^−^ fluxes, the CO_2_ leakage as well as the intrinsic fractionation by RubisCO [53], [54]. The C_i_ pool for calcification will mainly be controlled by the condition in the cytosol, which in turn is largely affected by the processes in the chloroplast. Discrimination of ^13^C during fixation will lead to ^13^CO_2_ efflux from the chloroplast, causing the cytosolic C_i_ pool to be enriched with ^13^CO_2_. If this ^13^CO_2_ is prevented from fast conversion to HCO_3_^−^ due to a lack of cytosolic CA activity, it could enter the calcifying vesicle by diffusion and be ‘trapped’ by the high pH resulting from proton pumping (Fig. 5). In fact, we do observe a higher ε_p_ (i.e. more ^13^CO_2_ can accumulate) and lower overall CA activities under elevated *p*CO_2_ (i.e. ^13^CO_2_ is not rapidly converted to HCO_3_^−^), which could have attributed to the opposing trends of ^13^C fractionation during organic and inorganic carbon production. To fully understand the intriguing interplay between these processes and their ^13^C fractionation, detailed measurements on the modes of C_i_ acquisition in *T. heimii* are needed.

The oxygen isotopic composition (δ^18^O) of calcite was also strongly affected in *T. heimii*, and increased by almost 6 ‰ over the investigated CO_2_ range (Fig. 3B). Even though biologically mediated, precipitation of calcite is an abiogenic process, which does not directly involve enzymatic reactions and thus mainly depends on the carbonate chemistry at the calcification site. Assuming negligible fractionation during the transport into the calcification vesicle, δ^18^O_calcite_ should therefore predominantly reflect the δ^18^O of the C_i_ species used for calcification. C_i_ species differ strongly in their δ^18^O values, ranging from lower values for CO_3_^2−^ (−4.7 ‰) and HCO_3_^−^ (2.1 ‰) to much higher values for CO_2_ (11.2 ‰) [24]. A previous study proposed a conceptual model to explain the δ^18^O dependence of *T. heimii* calcite and other unicellular planktonic calcifiers on seawater CO_3_^2−^ concentration (Fig. 3C) [9]. The authors attribute the negative slope between δ^18^O and [CO_3_^2−^] to an increased contribution of HCO_3_^−^ to the calcification vesicle. Also in our data, δ^18^O_calcite_ increases with increasing *p*CO_2_, starting from values close to the δ^18^O of CO_3_^2−^ towards those of HCO_3_^−^ (Fig. 3B). As argued above, however, the C_i_ pool in the calcifying vesicle may also be increasingly influenced by CO_2_, which is in line with the observed trends in δ^18^O_calcite_. Such a shift in C_i_ speciation may be an indication for a lowered intracellular pH, which in fact could be the reason for the hampered calcification under elevated *p*CO_2_ [55], [56].

Multiple genes associated to calcification have been described for *E. huxleyi* and include genes associated to the regulation of inorganic ions [39], [55]–[59]. Here we show that the expression of genes in *T. heimii* being involved in inorganic ion transport, in particular Ca^2+^ transport, decreased upon elevated *p*CO_2_ (Fig. 4; Fig. S3). This decrease in ion transport is in line with the observed decrease in calcification, which is comparable to observations in *E. huxleyi* [39], [59]. We also observed a strong CO_2_ dependent regulation of the vacuolar H^+^-ATPases (V-ATPase). These pumps play a key role in generating H^+^ gradients and membrane voltage, which drive multiple transport processes [57], [60]. As indicated from our data, H^+^-ATPases seem to play an important role in calcification in *T. heimii*, which is in agreement to observations for *E. huxleyi* and *Pleurochrysis carterae* [39], [59], [61]. Here we propose a conceptual model of calcification in *T. heimii*, which comprises some of the main processes described in this study (Fig. 5). Although many processes remain to be elucidated, this is a first step towards understanding the process of calcification in dinoflagellates.

### Paleo Proxies

The δ^18^O isotopic composition of *T. heimii* cysts has been used for the reconstruction of past temperatures [22], [62]. Indeed, δ^18^O changed linearly from about −1 to −4 ‰ with an increase in temperature from about 12 to 30°C. At the same time, however, pH decreased from about 8.4 to 7.9 in this study [22]. Hence, the observed changes in δ^18^O were most probably a result of both changes in temperature and seawater carbonate chemistry [see also 62]. Here we show remarkable changes in δ^18^O from about 0 to −5 ‰ with an increase in [CO_3_^2−^] from 50 to 260 µmol L^−1^, which is largely in agreement to an earlier study including a different *T. heimii* strain (Fig. 3C) [9]. Interestingly, the observed slopes of δ^18^O/[CO_3_^2−^] in both *T. heimii* strains are up to 10-fold steeper compared the coccolithophore *Calcidiscus leptoporus* and different foraminifera species [9], [25], [63]. Thus, the apparent ^18^O fractionation during calcification in *T. heimii* is much more sensitive to changes in [CO_3_^2−^] as compared to other key planktonic marine calcifiers. The steep slope and negative correlation between δ^18^O and [CO_3_^2−^] observed in both *T. heimii* strains suggests that the δ^18^O in *T. heimii* cysts may be a good candidate to serve as a proxy for past CO_3_^2−^ concentrations in ocean waters. This relationship may provide an ideal asset, especially when combined with different δ^18^O/[CO_3_^2−^] slopes observed in for instance coccolithophores, which will exclude confounding effects of additional environmental parameters such as temperature. Ultimately, this proxy could be further developed for reconstructing past atmospheric *p*CO_2_.

### Conclusion

We observed a strong reduction in growth rate and calcification of *T. heimii* under elevated *p*CO_2_. Although the function of calcification in *T. heimii* remains unresolved, it likely plays an important role in its ecological and evolutionary success. Acting on calcification as well as growth, ocean acidification may impose a great threat for *T. heimii*. Furthermore, the strong correlations between the stable isotope composition and carbonate chemistry suggest a great potential of *T. heimii* cysts to be used as paleo proxy for reconstructing seawater carbonate chemistry and ultimately past atmospheric *p*CO_2_.

## Supporting Information

Figure S1 **Population growth dynamics.** Population densities in each replicate over time in the (A) 150 µatm, (B) 380 µatm, (C) 750 µatm, and (D) 1400 µatm CO_2_ treatments. Lines indicate an exponential function fitted through the population densities (*n* = 8) of replicate 1 (black), 2 (grey) and 3 (white), with (A) 1: R^2^ = 0.98, p<0.0001, 2: R^2^ = 0.97, p<0.0001, and 3: R^2^ = 0.97, p<0.0001, (B) 1: R^2^ = 0.97, p<0.0001, 2: R^2^ = 0.97, p<0.0001, and 3: R^2^ = 0.92, p<0.0001, (C) 1: R^2^ = 0.92, p = 0.0007, 2: R^2^ = 0.96, p<0.0001, and 3: R^2^ = 0.97, p<0.0001, and (D) 1: R^2^ = 0.96, p<0.0001, 2: R^2^ = 0.95, p<0.0001, and 3: R^2^ = 0.91, p = 0.0002.
(EPS)

Figure S2 **Number of expressed genes.** Venn diagram of the number of expressed genes in the 150 µatm, 380 µatm, and 1400 µatm CO_2_ treatments.
(EPS)

Figure S3 **Distribution of expressed genes grouped according to KOG.** Values represent the number of genes expressed per KOG, relative to the total number of genes expressed in the respective treatment.
(EPS)

Table S1 **Carbonate chemistry at the start and end of the experiment.** Overview of *p*CO_2_, pH_NBS_, dissolved inorganic carbon (DIC), CO_2_ concentration in the water, total alkalinity (TA), and the seawater calcite saturation state Ω_calcite_. Values indicate mean ± SD (*n* = 3).
(DOCX)

Table S2 **Growth, elemental composition and calcification at the end of the experiment.** Overview of growth rate, POC production, carbon quota (TPC, POC, and PIC), PIC:POC ratio, and the number of completed cysts. Values indicate mean ± SD (*n* = 3).
(DOCX)

Table S3 **Overview of all expressed genes grouped according to KOG.**
(XLSX)

Table S4 **Overview of the number of readings for genes associated to ion transport and C_i_ acquisition.**
(XLSX)
