# Supplementary material for: Ocean Acidification Reduces Growth and Calcification in a Marine Dinoflagellate
Source: PLoS One. 2013 Jun 11;8(6):e65987. doi: 10.1371/journal.pone.0065987 (PMC3679017; doi:10.1371/journal.pone.0065987)
Supplement: Table S1 — Carbonate chemistry at the start and end of the experiment. Overview of pCO2, pHNBS, dissolved inorganic carbon (DIC), CO2 concentration in the water, total alkalinity (TA), and the seawater calcite saturation state Ωcalcite. Values indicate mean ± SD (n = 3). (DOCX) [file pone.0065987.s004.docx]

Table S1.

|  | CO_2_ | *p*CO_2_ | pH_NBS_ | DIC | CO_2_ | TA | Ω_calcite_ |
| --- | --- | --- | --- | --- | --- | --- | --- |
|  | treatment | (µatm) |  | (µmol L^-1^) | (µmol L^-1^) | (µmol L^-1^) |  |
| Start | 150 | 151 ± 1 | 8.52 ± 0.01 | 1,982 ± 7 | 5.8 ± 0.1 | 2,432 ± 11 | 7.2 ± 0.07 |
|  | 380 | 330 ± 4 | 8.24 ± 0.01 | 2,127 ± 2 | 12.7 ± 0.1 | 2,395 ± 2 | 4.4 ± 0.04 |
|  | 750 | 624 ± 4 | 8.00 ± 0.01 | 2,232 ± 17 | 24.0 ± 0.2 | 2,386 ± 18 | 2.7 ± 0.03 |
|  | 1400 | 1,203 ± 28 | 7.73 ± 0.01 | 2,291 ± 7 | 46.2 ± 1.1 | 2,348 ± 5 | 1.5 ± 0.03 |
| End | 150 | 243 ± 7 | 8.35 ± 0.01 | 2,041 ± 24 | 9.4 ± 0.3 | 2,443 ± 3 | 5.5 ± 0.01 |
|  | 380 | 506 ± 9 | 8.08 ± 0.01 | 2,181 ± 20 | 19.4 ± 0.3 | 2,462 ± 33 | 3.2 ± 0.03 |
|  | 750 | 914 ± 19 | 7.85 ± 0.01 | 2,286 ± 17 | 35.1 ± 0.7 | 2,467 ± 21 | 2.0 ± 0.06 |
|  | 1400 | 1,719 ± 8 | 7.59 ± 0.01 | 2,371 ± 10 | 66.1 ± 0.3 | 2,458 ± 6 | 1.2 ± 0.01 |
