# Supplementary material for: Ocean Acidification Reduces Growth and Calcification in a Marine Dinoflagellate
Source: PLoS One. 2013 Jun 11;8(6):e65987. doi: 10.1371/journal.pone.0065987 (PMC3679017; doi:10.1371/journal.pone.0065987)
Supplement: Table S2 — Growth, elemental composition and calcification at the end of the experiment. Overview of growth rate, POC production, carbon quota (TPC, POC, and PIC), PIC:POC ratio, and the number of completed cysts. Values indicate mean ± SD (n = 3). (DOCX) [file pone.0065987.s005.docx]

Table S2.

| CO_2_ | Growth | POC production | TPC | POC^*^ | PIC | PIC:POC | Completed |
| --- | --- | --- | --- | --- | --- | --- | --- |
| treatment | (d^-1^) | (pg cell^-1^ d^-1^) | (pg cell^-1^) | (pg cell^-1^) | (pg cell^-1^) |  | cysts (%) |
| 150 | 0.34 ± 0.04^a^ | 100 ± 8^a^ | 364 ± 13^†^ | 293 ± 10^†^ | 71 ± 11^a^ | 0.24 ± 0.04^ab^ | 97.5 ± 1.2^a^ |
| 380 | 0.31 ± 0.03^a^ | 93 ± 9^a^ | 390 ± 14^†^ | 303 ± 26^†^ | 87 ± 12^b^ | 0.29 ± 0.06^a^ | 94.6 ± 3.7^a^ |
| 750 | 0.28 ± 0.03^a^ | 89 ± 9^a^ | 379 ± 7^†^ | 327 ± 70^†^ | 60 ± 10^a^ | 0.19 ± 0.03^b^ | 67.8 ± 4.4^b^ |
| 1400 | 0.16 ± 0.02^b^ | 57 ± 4^b^ | 379 ± 5^†^ | 349 ± 30^†^ | 37 ± 2^c^ | 0.11 ± 0.01^c^ | 20.3 ± 3.1^c^ |

^†^ No significant difference between the treatments (ANOVA, P>0.05); ^a,b,c^ Significant differences between treatments (ANOVA, P<0.05).
